# Microwave-Assisted Extraction Coupled to HPLC-UV Combined with Chemometrics for the Determination of Bioactive Compounds in Pistachio Nuts and the Guarantee of Quality and Authenticity

**DOI:** 10.3390/molecules27041435

**Published:** 2022-02-21

**Authors:** Natasa P. Kalogiouri, Petros D. Mitsikaris, Athanasios N. Papadopoulos, Victoria F. Samanidou

**Affiliations:** 1 Laboratory of Analytical Chemistry, Department of Chemistry, Aristotle University of Thessaloniki, 54124 Thessaloniki, Greece; kalogiourin@chem.auth.gr; 2 Laboratory of Chemical Biology, Department of Nutritional Sciences and Dietetics, International Hellenic University, Sindos, 57400 Thessaloniki, Greece; petrosmitsikaris@gmail.com (P.D.M.); papadnas@ihu.gr (A.N.P.)

**Keywords:** microwave-assisted extraction, tocopherols, phenolics, flavonoids, authenticity, HPLC-UV

## Abstract

Two novel microwave-assisted extraction (MAE) methods were developed for the isolation of phenols and tocopherols from pistachio nuts. The extracts were analyzed by reversed-phase high-pressure liquid chromatography coupled with a UV detector (RP-HPLC-UV). In total, eighteen pistachio samples, originating from Greece and Turkey, were analyzed and thirteen phenolic compounds, as well as α-tocopherol, (β + γ)-tocopherol, and δ-tocopherol, were identified. The analytical methods were validated and presented good linearity (r^2^ > 0.990) and a high recovery rate over the range of 82.4 to 95.3% for phenols, and 93.1 to 96.4% for tocopherols. Repeatablility was calculated over the range 1.8–5.8%RSD for intra-day experiments, and reproducibility over the range 3.2–9.4%RSD for inter-day experiments, respectively. Principal component analysis (PCA) was employed to analyze the differences between the concentrations of the bioactive compounds with respect to geographical origin, while agglomerative hierarchical clustering (AHC) was used to cluster the samples based on their similarity and according to the geographical origin.

## 1. Introduction

Nuts are appreciated for their distinctive taste and beneficial health properties. Various clinical and epidemiological studies have demonstrated a direct correlation between the consumption of nuts and numerous improvements in different health markers, such as cholesterol levels [1], glycemic control [2], and waist circumference [3]. The pistachio nut (*Pistacia vera* L.) is a prominent member of the nut family and is valued globally for both its sensory attributes and nutritional value.

Several studies have already displayed the cardioprotective, antioxidant, and anti-inflammatory properties of pistachios [4,5,6,7,8]. The health properties of pistachios are related to their favorable macro- and micronutrient profile. Pistachios have a rich phytochemical content; in particular, they are rich in phenolic compounds and tocopherols, which are two characteristic classes of chemical compounds with proven antioxidant effects [9,10]. According to the literature, the content of both phenols and tocopherols is affected by numerous factors, including plant genetics, variety, geographical origin, pre- and post-harvest factors, and climate conditions [11,12]. All of the aforementioned factors could positively or negatively affect the amounts of these bioactive constituents, hence directly affecting the quality of the nut. The analysis of pistachios could provide useful information in terms of evaluating and differentiating between different cultivars and geographical origins. For this reason, developing and optimizing rapid, easy-to-apply, and widely applicable analytical methods could ensure that nut products hold to a high standard of quality and also protect against adulteration incidents [13].

The isolation and determination of bioactive constituents in food products contains challenges in several distinct steps of the analytical procedure. Sample preparation is pivotal, since it is of the utmost importance to ensure that as little as possible of the targeted substances are lost during the process. There are a handful of protocols that have been introduced to the literature, though most of them propose the use of large volumes of toxic organic solvents [14]. The recent trends in sample preparation necessitate the development of extraction protocols that are compatible with sustainable green chemistry processes [15,16,17]. Ultrasound-assisted extraction has been successfully applied in the extraction of phenolics and tocopherols using methanol–water mixtures as extraction solvents [18,19]. Microwave-assisted extraction (MAE) is an automated green extraction process that enables the isolation of the target analytes with short times, dramatically decreases solvent consumption, and improves sample throughput [20]. MAE has been widely used for the isolation of functional compounds from plant matrices [19,21,22]; however, only a few works have investigated the use of green solvents such as ethanol–water mixtures, proposing them to be effective solvents that allow for safe extraction operations [17]. This gap in the literature has to be filled. The determination of phenols and tocopherols is commonly achieved through high-pressure liquid chromatography (HPLC) coupled to UV, photodiode arrays (DAD), mass spectrometric (MS) detectors, or a combination of the above [23,24,25,26,27,28,29,30].

The further coupling of the analytical methods with exploratory and discriminatory chemometric techniques allows for an in-depth interpretation of the results in the fields of food authenticity and traceability. Authenticity issues are multivariate, covering different aspects such as characterization, mislabeling, and adulteration. The development of chemometric models enables the extraction of useful information from food authenticity studies, enabling the discrimination of the samples according to their variety, geographical origin, and type of cultivar, among others [31,32].

This study reports, for the first time, the effective use of MAE in the isolation of phenolic compounds and tocopherols from pistachio nuts and pistachio oils, respectively, and their further determination by HPLC-UV. The phenolic and tocopherol content of *Pistacia vera* L. originating from Greece and Turkey was examined for the first time, and principal component analysis (PCA) and agglomerative hierarchical clustering (AHC) were used in order to explore similarities between the samples originating from different geographical regions.

## 2. Results

### 2.1. Method Validation

#### 2.1.1. Validation Results of the MAE-HPLC-UV Method for the Determination of Phenolic Compounds

The analytical performance of the MAE-HPLC-UV method was evaluated after measuring its trueness and precision, as well as calculating its linearity, limits of detection (LODs), and limits of quantification (LOQs). The validation results are presented in the Supplementary Materials in Table S1, and as it is observed that the coefficients of determination ranged between 0.991 and 0.997, showing a good linearity for all the analytes. The LODs and LOQs ranged between 0.10 and 0.50 μg/g and 0.20 and 1.80 μg/g, respectively. The RSD% of the within-day (n = 6) and between-day (n = 3 × 3) assays was lower than 5.8 and 8.8, respectively, presenting an adequate precision. The trueness was evaluated by means of relative percentage of recovery (%R) at the lowest, medium, and highest concentration level (0.5, 5, 10 µg/g) and ranged between 83.2 and 95.3% (for the within-day assay (n = 6), as presented in Table S2) and between 82.4 and 94.8% (for the between-day assay (n = 3 × 3), as presented in Table S3).

#### 2.1.2. Validation Results of the MAE-HPLC-UV Method for the Determination of Tocopherols

Table S4 presents the validation results of the MAE-HPLC-UV method for the determination of tocopherols. The calibration curves were linear in the entire working range (5–50 μg/g). The LODs and LOQs ranged from 0.10 to 0.30 and 0.30 to 0.90, respectively. The precision was good, as the RSD% of the within-day (n = 6) and between-day assays (n = 3 × 3) was lower than 5.3 and 8.7, respectively. Trueness was assessed by means of the relative percentage of recovery at the lowest, medium, and highest concentration levels (0.5, 5, 10 µg/g) and ranged between 93.1 and 96.8% for the within-day assay (n = 6), as shown in Table S5, and between 93.3 and 96.4% for the between-day assay (n = 3 × 3), as shown in Table S6.

### 2.2. Pistachio Nuts Analysis

#### 2.2.1. Identification and Quantification Results of Phenolics

**Figure 1:**
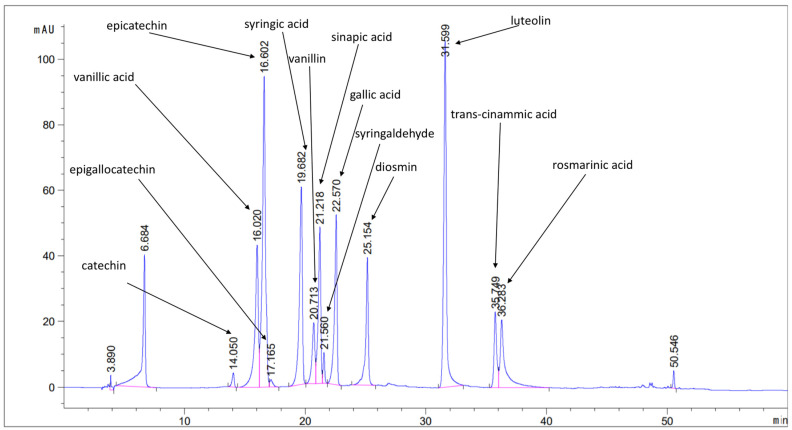
Characteristic chromatogram of a pistachio sample spiked at 5 μg/g and monitored at 280 nm.

The developed methods were applied in the analysis of phenols and tocopherols in eighteen pistachio samples produced in Turkey and Greece. In total, thirteen phenolic compounds were determined. Catechin, diosmin, epicatechin, epigallocatechin, gallic acid, luteolin, rosmarinic acid, sinapic acid, syringaldehyde, syringic acid, trans-cinnamic acid, vanillic acid, and vanillin were all determined. Figure 1 shows a characteristic chromatogram of a pistachio sample spiked at 5 µg/g with the target analytes. The concentration ranges of all of the determined analytes, as well as the mean values for each sample, are presented in Table 1. All samples were analyzed in triplicate (n = 3, µg/g ±SD).

The concentration ranges of the determined phenolic compounds are in accordance with the current literature [33,34]. According to Table 2, diosmin, epigallocatechin, and rosmarinic acid were not detected in the pistachio nuts originating from Turkey. On the contrary, the average concentration of diosmin, epigallocatechin, and rosmarinic acid was equal to 24.73, 6.51, and 8.55 μg/g, respectively, in the pistachio nuts produced in Greece. Gallic acid exhibited the highest concentration in all of the samples. The determined phenolic compounds have been associated with numerous health-promoting effects, such as antioxidant, anti-inflammatory, antimicrobial, anti-diabetic, anti-mutagenic, and cytoprotective effects, which corroborate the beneficial health effects associated with pistachios’ consumption.

#### 2.2.2. Determination of Tocopherols

**Figure 2:**
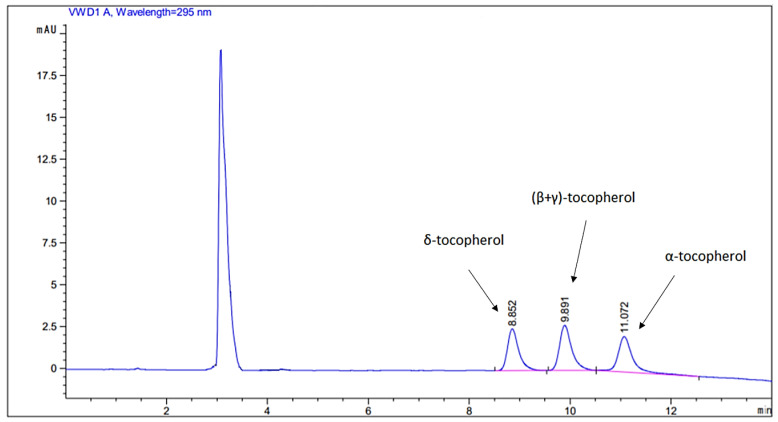
Characteristic chromatogram of a pistachio sample spiked at 5 μg/g and monitored at 295 nm.

The separation of tocopherols was achieved within 12 min. The gradient elution program enabled the separation of δ-tocopherol (Rt = 8.9 min) and α-tocopherol (Rt = 11.1 min). β- and γ-tocopherol co-eluted (Rt = 9.8 min) and were analyzed together, hereafter referred to as (β + γ)-tocopherol [35]. A representative chromatogram of a 5 µg/g standard solution mixture is shown in Figure 2. Each pistachio oil was analyzed in triplicate. The quantification ranges and the mean values (±SD) are presented in Table 2. The determined quantification ranges were in accordance with previously reported data [36]. According to the results, α-tocopherol, (β + γ)-tocopherol, and δ-tocopherol existed within the samples in relatively high concentrations. The highest average concentrations were observed in the mixture of (β + γ)-tocopherol, equal to 115.44 µg/g in the Aegina type pistachios from Greece, and 129.12 µg/g in the Antep type pistachios from Turkey. Lower α-tocopherol concentration ranges were determined in pistachios produced in Turkey (13.00–25.00 µg/g) as compared to the pistachios produced in Greece (36.00–78.00 µg/g). On the other hand, the average concentration of δ-tocopherol was higher in the pistachios originating from Turkey (24.12 µg/g) as compared to those produced in Greece (10.99 µg/g). 

### 2.3. Chemometrics

#### 2.3.1. Principal Component Analysis

**Figure 3:**
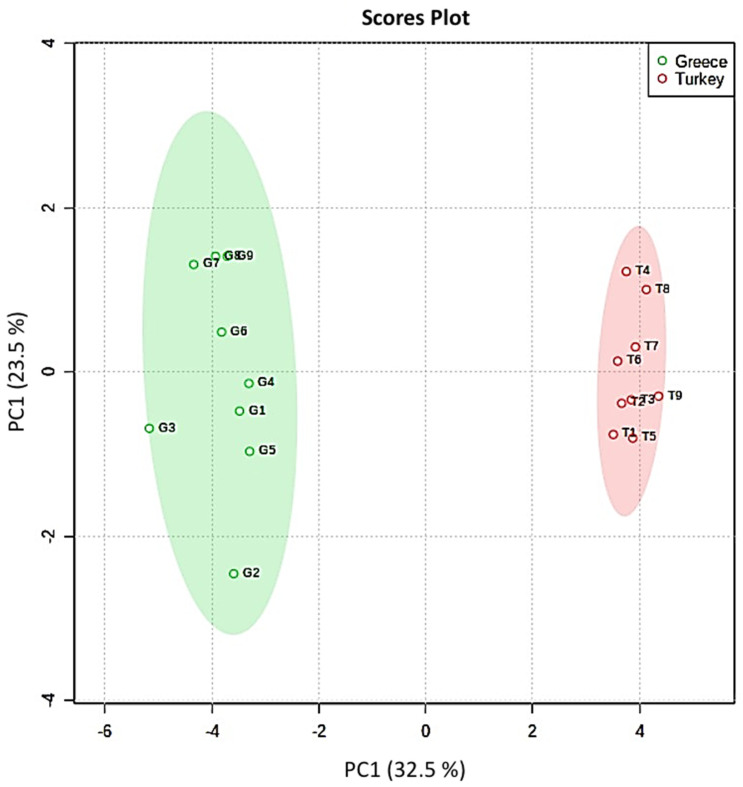
PCA scores plot illustrating the clustering between pistachio nuts originating from Greece and Turkey.

PCA was employed to explore distribution of the samples and formation of groups on the basis of the concentrations of the determined bioactive compounds. The data matrix consisted of eighteen pistachio samples originating from Greece and Turkey and sixteen features (the concentration results of phenolics and tocopherols). The MetaboAnalyst package was used for PCA, and the data matrix was auto-scaled [37]. Figure 3 presents the score plot and it is observed that the samples clustered into two individual groups according to the country of production. The pistachio nuts originating from Greece were grouped into the green ellipse, and those produced in Turkey were grouped into the red ellipse. The first two principal components (PCs) explained 55% of the total variance, establishing two individual groups of samples according to the country of production. The biplot presented in Figure S1 graphically shows, using the loadings, the effects of the variables in each PC.

#### 2.3.2. Agglomerative Hierarchical Clustering 

**Figure 4:**
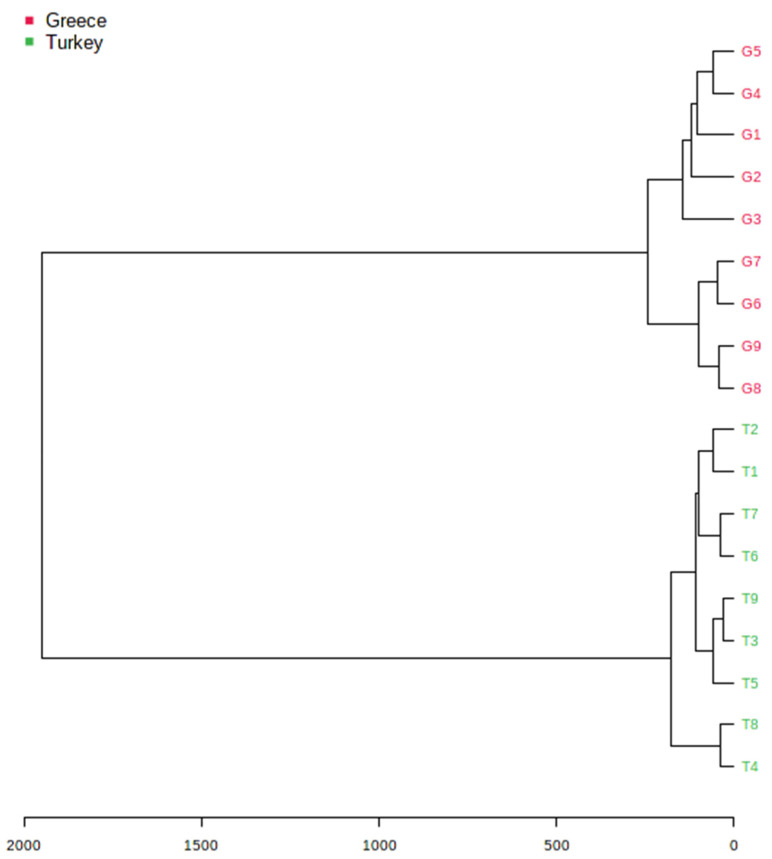
AHCdendrogram of pistachios originating from Greece (G1–G9) and Turkey (T1–T9) clustered into two major groups.

AHC was employed as a cluster analysis method to build a hierarchy of clusters in a tree diagram (also known as a dendrogram) to identify samples that present similarities and to group all of the objects that share similar characteristics into a large cluster [37]. The dendrogram presented in Figure 4 shows that the observations clustered into two major groups, one which was comprised of all of the pistachio samples produced in Greece (G1–G9), and a second one which comprised all of the samples originating from Turkey (T1–T9).

## 3. Materials and Methods

### 3.1. Chemicals and Reagents

HPLC-grade Methanol (MeOH) and HPLC-grade acetonitrile (ACN) were purchased from Carl Roth (Carlsruhe, Germany). Isopropanol (IPA) was purchased from Panreac-AppliChem (Darmstadt, Germany). Acetic acid (99%) was purchased from Sigma-Aldrich (Steinheim, Germany). To obtain ultrapure water, a Milli-Q purification system (Millipore, Bedford, MA, USA) was utilized. Analytical standards of catechin (98%), diosmin (97%), epicatechin (97%), epigallocatechin (98%), gallic acid (98%), trans-cinnamic acid (97%), syringaldehyde (98%), rosmarinic acid (98%), sinapic acid (95%), syringic acid (95%), vanillic acid (97%), luteolin (98%), vanillin (98%), α-tocopherol (96%), β-tocopherol (96%), γ-tocopherol (96%), and δ-tocopherol (96%) were acquired from Sigma-Aldrich (Steinheim, Germany). Stock standard solutions at 1000 μg/g were prepared for each analyte in methanol and stored at –20 °C. 

### 3.2. Sampling and Pre-Treatment

Eighteen pistachio samples (approximately 300 g each) originating from Greece (Aegina type) and Turkey (Antep type) were collected from local producers and importers. These samples were available in the Greek market in 2020. The pistachio seeds were separated from the endocarps, and each sample of 300 g was homogenized and then crushed using a mortar and pestle. The samples were lyophilized for 24 h. The lyophilized samples were stored at –20 °C until analysis. 

### 3.3. Instrumentation

A 1220 Infinity HPLC-UV system from Agilent Technologies (Santa Clara, CA, USA) was used for the chromatographic analysis of the phenolic compounds and tocopherols. The HPLC system consisted of a degasser, a column oven, a manual injector, and a UV detector. For monitoring the analysis, the OpenLAB software (Agilent Technologies, Santa Clara, CA, USA) was used with the Method and Run Control package. For peak identification and data processing, the Data Analysis software package (Agilent Technologies, Santa Clara, CA, USA) was used. QMax RR syringe filters (0.22 µm Nylon; and 0.22 PTFE) were acquired from Frisenette ApS (Knebel, Denmark) to filter the samples prior to analysis. For agitation, a vortex mixer from VELP Scientifica (Usmate Velate, Italy) was used. Centrifugation was carried out using a 3-16PK centrifuge system from Sigma (Osterode am Harz, Germany). For the green extractions, a MARS X Model 1000 microwave oven (CEM, Matthews, NC, USA) equipped with a 14-position holder, PTFE vessels, and a stirring mechanism was used.

### 3.4. Microwave-Assisted Extraction

For the extraction of the phenolics, a modified MAE protocol previously reported by Gallo et al. [38] was used. Extractions were performed at 200 W and 50 °C, and a magnetic stirring rod was added into each vessel. Approximately 1 g of each lyophilized sample was extracted with a mixture of ethanol and water (50:50 *v/v*, 5 mL), at 200 W using magnetic stirring at 50% of nominal power and a temperature of 50 °C for 15 min. The extract was collected and centrifuged for 10 min at 12,000 rpm. Then, 0.5 mL of the supernatant was collected, dried under nitrogen air, and reconstituted in 0.5 mL of MeOH–mobile phase A (10:90, *v/v*) and filtered through 0.22 μm nylon syringe filters. Finally, 20 μL was injected into the chromatographic system. 

Prior to the isolation of the tocopherols, a MAE protocol was employed for oil extraction [39]. Approximately 1 g of lyophilized sample was extracted using a mixture of acetone (2:1 *v/v*, 5 mL), at a microwave power of 420 W and temperature of 80 °C for 30 min. After extraction, the solvent was distilled with a rotary evaporator at 60 °C. The oil was collected. For the extraction of tocopherols, a modified version of a previously reported protocol [40] was applied using 20 mg of oil dissolved in 1 mL of ethanol in a 2 mL Eppendorf tube. The mixture was vortexed for 1 min at 3000 rpm and then centrifuged for 10 min at 12,000 rpm. The supernatant was collected and filtered through 0.22 µm PTFE syringe filters and 20 µL was injected into the HPLC system.

### 3.5. Chromatographic Analysis

The analysis of the polyphenols was conducted at 280 nm in a Macherey-Nagel (Düren, Nordrhein-Westfalen, Germany) Nucleosil RP-18 analytical column (250 mm × 4.6 mm, 5 μm particle size). The mobile phase comprised of 1% *v/v* formic acid in water (A) and ACN 0.5% (B), at a 1 mL/min flow rate. The column was thermostatically controlled at 30 °C. The flow rate was set at 1 mL/min and each chromatographic run had a duration of 60 min. The chromatographic program started with 5% B, gradually increased to 20% during the first 15 min, then to 50% until 40 min, then from 50% to 90% over the following 5 min, and then kept stable until 50 min. Then, the organic phase decreased to 5% between 50 and 55 min, and then remained constant for the next 5 min. 

For the analysis of the tocopherols, a Kromasil RP-18 analytical column (125 mm × 4.6 mm, 5 µm particle size) was acquired from Macherey-Nagel. The UV wavelength was set to 295 nm. The mobile phase consisted of MeOH (A) and ACN (B). The column was thermostatically controlled at 30 °C, and the flow rate was set to 1 mL/min. Each chromatographic run lasted for 15 min. The elution program started with 50% B and remained stable for 7 min, then decreased to 0% over the following 5 min and remained stable for 3 min. For the identification of the compounds, the RTs of the peaks in the extracts were compared with the RTs of their respective standards. Spiked extracts of different concentrations (e.g., 0.5, 5, 10, and 20 μg/g), depending on the peak intensities of the real samples, were also injected into the chromatographic system and the RTs were matched with the corresponding neat extracts to verify their presence.

### 3.6. Method Validation 

The developed methods were validated in terms of linearity, trueness, precision, limits of detection (LODs), and limits of quantification (LOQs). To assess linearity, calibration curves were constructed over the range 0.5–20 µg/g for phenolic analytes and over the range 5–50 µg/g for tocopherols using 8 concentration points. The LODs were equal to 3.3 multiplied by the signal-to-noise ratio (S/N), while LOQs were equal to 10 multiplied by the S/N ratio. Trueness and precision were evaluated using a real spike at three concentration levels: 0.5, 10, and 20 µg/g for the phenolic compounds, and 5, 25, and 50 µg/g for the tocopherols [40]. Within-day precision (repeatability) was assessed using six replicate spiked samples (n = 6), while between-day precision (reproducibility) was estimated by analyzing spiked samples in triplicate over three days (n = 3 × 3). 

### 3.7. Chemometric Analysis

PCA is an unsupervised technique used for exploratory chemometric data analysis. PCA is a dimensionality reduction method that computes the PCs and uses them make changes in a dataset. AHC was used to cluster the samples according to their resemblance. PCA and AHC were executed in R using the MetaboAnalyst 5.0 package [41].

## 4. Conclusions

Two MAE-HPLC-UV methods were proposed as innovative green approaches for the analysis of phenolic compounds and tocopherols in pistachio nuts. Eighteen pistachio samples produced in Turkey and Greece were analyzed, and the phenolic compounds catechin, diosmin, epicatechin, epigallocatechin, gallic acid, luteolin, rosmarinic acid, sinapic acid, syringaldehyde, syringic acid, trans-cinammic acid, vanillic acid, and vanillin were determined. Ιn pistachio oils, α-tocopherol, δ-tocopherol, and the sum of β- and γ-tocopherols were determined. The quantification results were analyzed using PCA, and the samples were distributed into two individual groups according to the geographical origin. The first two PCs explained 56% of the total variance. Furthermore, an AHCdendrogram was also created, which also clustered the samples, on the basis of their similarities, into two major groups. Overall, two green extraction methods are suggested for the isolation of phenolics and tocopherols, supporting the idea that these compounds could be used as markers for the authentication of pistachios. 

## Figures and Tables

**Table 1:** Concentration ranges and mean values of the phenolic analytes determined in pistachios originating from Greece and Turkey.

	Greek Pistachios		Turkish Pistachios	
Compound	Concentration Range (μg/g)	Mean Value (±SD, μg/g)	Concentration Range (μg/g)	Mean Value (±SD, μg/g)
catechin	25.21–46.80	37.08 ± 5.42	5.96–22.00	13.02 ± 4.00
diosmin	22.60–29.48	24.73 ± 2.04	ND	ND
epicatechin	78.20–124.58	90.12 ± 13.28	ND–5.64	3.21 ± 1.95
epigallocatechin	ND–12.60	6.51 ± 4.20	ND	ND
gallic acid	225.77–274.00	249.83 ± 14.25	122.00–188.00	151.23 ± 21.74
luteolin	12.97–29.74	21.20 ± 4.82	ND–5.86	4.27 ± 1.63
rosmarinic acid	4.32–14.60	8.55 ± 3.32	ND	ND
sinapic acid	39.14–66.40	55.64 ± 7.86	ND–2.21	0.54 ± 0.80
syringaldehyde	15.12–23.79	20.40 ± 3.03	1.85–12.40	7.13 ± 3.02
syringic acid	12.60–15.60	14.07 ± 1.05	ND–2.45	1.32 ± 1.01
trans-cinammic acid	ND–0.88	0.18 ± 0.35	1.05–2.24	1.67 ± 0.34
vanillic acid	ND	ND	3.21–5.32	4.42 ± 0.61
vanillin	3.22–8.32	6.27 ± 1.49	1.06–3.33	1.86 ± 0.71

**Table 2:** Concentration ranges and mean values of the tocopherols determined in pistachios originating from Greece and Turkey.

Tocopherols	Greek Pistachios		Turkish Pistachios	
	Concentration Range (μg/g)	Mean Value (±SD, μg/g)	Concentration Range (μg/g)	Mean Value (±SD, μg/g)
α-tocopherol	36.00–78.00	57.77 ± 11.99	13.00–25.00	16.56 ± 3.50
(β + γ)-tocopherol	78.00–152.00	115.44 ± 23.97	105.09–156.00	129.12 ± 17.56
δ-tocopherol	10.99–18.90	15.35 ± 2.46	20.80–27.40	24.12 ± 2.36

## Data Availability

Not applicable.

## Supplementary Materials

The following supporting information can be downloaded online. Table S1: Analytical parameters of the MAE-HPLC-UV method for the determination of phenolics; Table S2: Intra-day recoveries and repeatability results of the MAE-HPLC-UV for the determination of phenolics; Table S3: Inter-day recoveries (%R) and reproducibility results of the MAE-HPLC-UV for the determination of phenolics; Table S4: Analytical parameters of the MAE-HPLC-UV for the determination of tocopherols; Table S5: Inter-day recoveries and repeatability results of the MAE-HPLC-UV method for the determination of tocopherols; Table S6: Inter-day recoveries (%R) and reproducibility results of the MAE-HPLC-UV for the determination of tocopherols; Figure S1: PCA biplot.

## Sample Availability

Samples of the compounds are not available from the authors.
