# Supplementary material for: Microwave-Assisted Extraction Coupled to HPLC-UV Combined with Chemometrics for the Determination of Bioactive Compounds in Pistachio Nuts and the Guarantee of Quality and Authenticity
Source: Molecules. 2022 Feb 21;27(4):1435. doi: 10.3390/molecules27041435 (PMC8875453; doi:10.3390/molecules27041435)
Supplement: Supplementary file 1 [file molecules-27-01435-s001.zip › molecules-1603781-supplementary.pdf]

Supplementary Material

Microwave-Assisted Extraction Coupled to HPLC-UV Combined with Chemometrics for the Determination of Bioactive Compounds in Pistachio nuts and the Guarantee of Quality and Authenticity

Natasa P. Kalogiouri, Petros Mitsikaris, Athanasios N. Papadopoulos and Victoria F. Samanidou

Table S1. Analytical parameters of the MAE-HPLC-UV method for the determination of phenolics

Compound	Calibration equation	Linear range (μg/g)	r^2	LOD (μg/g)	LOQ (μg/g)
catechin	y = 20.04x + 5.88	2 - 20	0.995	0.40	1.20
diosmin	y = 14.05 x – 0.48	2 - 20	0.994	0.60	1.80
epicatechin	y = 18.94x + 3.55	2 - 20	0.994	0.40	1.20
epigallocatechin	y = 64.25x – 5.32	2 - 20	0.997	0.50	1.50
gallic acid	y = 36.34x + 0.33	0.5 - 20	0.995	0.07	0.20
luteolin	y = 38.44x + 1.08	2 - 20	0.996	0.50	1.50
rosmarinic acid	y = 64.34x + 0.56	0.5 - 20	0.994	0.10	0.30
sinapic acid	y = 26.34x + 1.88	1 - 20	0.996	0.30	0.90
syringaldehyde	y = 34.75x – 4.22	1 - 20	0.993	0.30	0.90
syringic acid	y = 41.87x – 1.55	0.5 - 20	0.995	0.10	0.30
trans-cinnamic acid	y = 67.35x + 3.45	1 - 20	0.997	0.10	0.30
vanillic acid	y = 68.78x + 4.14	1 - 20	0.996	0.30	0.90
vanillin	y = 20.47x + 3.75	0.5 - 20	0.991	0.10	0.30

LOD: limit of detection, LOQ: limit of quantitation

Table S2. Intra-day recoveries and repeatability results of the MAE-HPLC-UV for the determination of phenolics

Compound	Low Concentration (%R, n = 6)	%RSD	Medium Concentration (%R, n = 6)	%RSD	High Concentration (%R, n = 6)	%RSD
catechin	91.2	3.9	96.2	3.8	95.3	2.2
diosmin	90.5	3.6	91.5	4.2	94.4	1.8
epicatechin	86.7	5.2	89.8	3.8	92.8	2.4
epigallocatechin	91.1	4.4	92.2	3.9	89.7	5.1
gallic acid	89.4	3.9	88.9	5.7	90.5	3.9
luteolin	91.7	4.1	85.2	5.3	91.6	3.3
rosmarinic acid	88.8	2.8	91.4	5.4	92.7	4.8
sinapic acid	86.2	5.8	93.3	3.9	94.5	2.2
syringaldehyde	88.5	3.5	92.4	2.9	89.8	4.1
syringic acid	91.5	2.2	89.5	4.4	95.3	5.3
trans-cinnamic acid	83.2	4.1	85.2	3.9	92.2	2.5
vanillic acid	84.7	3.8	90.8	5.1	94.5	5.5
vanillin	92.8	4.8	91.1	4.7	93.2	4.5

Table S3. Inter-day recoveries (%R) and reproducibility results of the MAE-HPLC-UV for the determination of phenolics

Compound	Low Concentration (%R, n = 3 x 3)	%RSD	Medium Concentration (%R, n = 3 x 3)	%RSD	High Concentration (%R, n = 3 x 3)	%RSD
catechin	89.3	3.9	91.8	7.4	91.5	4.8
diosmin	82.4	7.5	91.7	7.9	90.8	7.3
epicatechin	85.2	6.5	92.8	6.5	93.1	6.2
epigallocatechin	82.7	6.2	94.5	5.8	89.4	7.1
gallic acid	89.8	8.4	91.9	7.4	93.8	5.4
luteolin	91.4	8.1	92.8	8.8	94.8	3.2
rosmarinic acid	93.6	6.1	95.9	4.2	93.1	5.2
sinapic acid	92.2	5.4	92.7	4.7	92.8	4.4
syringaldehyde	89.5	5.5	93.5	8.1	93.3	6.1
syringic acid	91.9	8.3	92.9	6.3	91.9	5.6
trans-cinnamic acid	94.5	7.9	89.9	7.9	93.7	6.2
vanillic acid	94.4	8.4	93.8	9.4	91.2	7.9
vanillin	92.2	6.7	91.7	6.9	93.3	8.3

Table S4. Analytical parameters of the MAE-HPLC-UV for the determination of tocopherols

Compound	Calibration equation	Linear range (μg/g)	r^2	LOD (μg/g)	LOQ (μg/g)
α-tocopherol	y = 6.21x – 1.33	5 – 50	0.997	0.30	0.90
(β+γ)-tocopherol	y = 7.48x + 0.16	5 – 50	0.995	0.10	0.30
δ-tocopherol	y = 6.06x + 0.49	5 - 50	0.998	0.20	0.60

LOD: limit of detection, LOQ: limit of quantitation

Table S5. Inter-day recoveries and repeatability results of the MAE-HPLC-UV method for the determination of tocopherols

**Compound**	**Low Concentration (%R, n = 6)**	**%RSD**	**Medium Concentration (%R, n = 6)**	**%RSD**	**High Concentration (%R, n = 6)**	**%RSD**
α-tocopherol	93.1	2.7	95.1	3.6	96.8	4.7
(β+γ)-tocopherol	95.7	3.6	96.6	4.4	95.9	5.3
δ-tocopherol	96.8	5.2	96.6	4.3	94.9	4.2

Table S6. Inter-day recoveries (%R) and reproducibility results of the MAE-HPLC-UV for the determination of tocopherols

**Compound**	**Low Concentration (%R, n = 3 x 3)**	**%RSD**	**Medium Concentration (%R, n = 3 x 3)**	**%RSD**	**High Concentration (%R, n = 3 x 3)**	**%RSD**
α-tocopherol	93.3	6.3	95.5	7.9	95.7	6.4
(β+γ)-tocopherol	95.5	8.7	96.2	8.1	96.1	7.2
δ-tocopherol	95.8	5.5	96.6	6.9	96.4	5.1

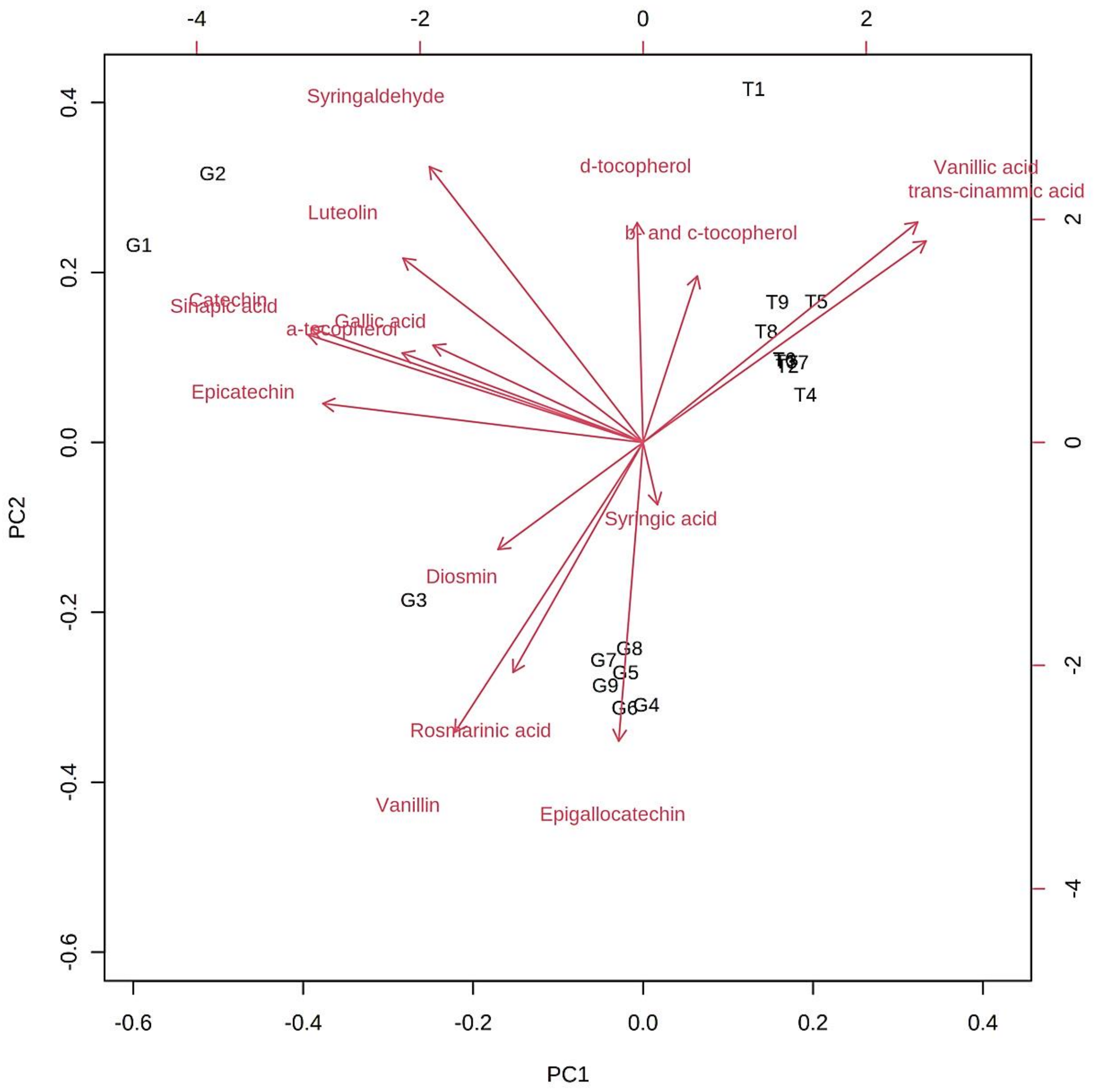

Figure S1. PCA biplot
